# Acute Renal Failure and Jaundice without Methemoglobinemia in a Patient with Phenazopyridine Overdose: Case Report and Review of the Literature

**DOI:** 10.1155/2014/845372

**Published:** 2014-03-05

**Authors:** Ian Holmes, Nathaniel Berman, Vinicius Domingues

**Affiliations:** New York Presbyterian Hospital/Weill Cornell Medical College, New York, NY 10065, USA

## Abstract

Phenazopyridine is a commonly used urinary analgesic available throughout the United States. Ingestion of large quantities can lead to methemoglobinemia, hemolytic anemia, jaundice, and acute renal failure. We report a case of a 78-year-old male with previously normal renal function who developed acute renal failure and jaundice without methemoglobinemia or hyperbilirubinemia after taking nearly 8 g of phenazopyridine over the course of 4 days. Initially presenting with oliguria, the urine output began to increase by day 2 of his admission, and the creatinine peaked 11 days after he began taking phenazopyridine, and he was discharged safely soon after. To our knowledge, this is the first such case of renal failure and jaundice without methemoglobinemia or hemolytic anemia in an adult patient with normal renal function.

## 1. Introduction

Phenazopyridine is a commonly prescribed urinary analgesic which has been in use since the 1920s [1]. Its use is associated with gastrointestinal discomfort and orange discoloration of urine [2]. Rarely, patients have developed pigment stones due to the red azo dye which is part of the drug's formulation [1]. In large doses, phenazopyridine has been reported to cause renal failure, methemoglobinemia, skin pigmentation, and hemolytic anemia [2–7]. These findings often occur together, although isolated renal failure has been reported in pediatric patients and in patients with underlying renal disease [4, 7, 8]. We report a case of anuric renal failure and jaundice due to phenazopyridine without hyperbilirubinemia, methemoglobinemia, or hemolytic anemia in a patient without underlying renal disease. Previous case reports of phenazopyridine-associated kidney injury have either been in pediatric patients or in patients with underlying renal disease; to our knowledge, this is the first such case in a patient with normal renal function [3].

## 2. Case Report

A 78-year-old male physician with no prior renal disease presented to the emergency department with anuria. Five days prior to presentation, he had developed dysuria and urinary frequency and started himself on levofloxacin and phenazopyridine. Over the course of four days, he had taken 39 200 mg tabs of phenazopyridine (approximately 7.8 g). One day prior to admission, he noted that his urine output had ceased despite increased oral intake and decided to present to the emergency department.

On presentation, the patient was hypotensive to 70/40 with a heart rate of 91 bpm but had no orthostatic symptoms. He received four liters of normal saline with no improvement in urine output, although his systolic blood pressure increased to 130 mmHg. Additionally, he was found to be jaundiced with anicteric sclera. Creatinine in the emergency department was 6.16 mg/dL (increased from his baseline of 1.0 mg/dL several weeks previously) while BUN was 42 mg/dL. Urinalysis revealed a bright orange appearance with pigmented casts. A renal ultrasound showed no evidence of obstruction.

The patient was treated conservatively following fluid resuscitation. Given the recent history of significant phenazopyridine intake, a biopsy was not performed. Urine output returned on the second day of admission, with the patient producing over 500 mL of orange urine. By the eighth day he was producing over two liters of urine per day. Urine electrolytes were notable for a fractional excretion of sodium of 3.95%. His creatinine rose steadily before peaking at 10.7 mg/dL 11 days following his first ingestion of phenazopyridine. The patient never developed signs of methemoglobinemia or hemolytic anemia; his methemoglobin never rose above 2.1% while LDH, haptoglobin, and reticulocyte counts were within normal limits. Total bilirubin peaked at 1.5 mg/dL. The patient maintained brisk diuresis and was discharged ten days after admission.

## 3. Discussion

Phenazopyridine is a commonly used analgesic with a topical effect on the urinary tract mucosa. It is available without a prescription and although usually safe in small doses it can cause various toxicities in large doses [7]. It is commonly linked to gastrointestinal discomfort and orange urine but has also been reported to cause Heinz body hemolytic anemia, renal failure, methemoglobinemia, and skin discoloration [2]. 90% of the consumed dose is excreted in the urine within the first 24 hours following ingestion [1, 2]. The majority of this is comprised of the parent drug, although toxic metabolites *p-*aminophenol, *N-*acetyl-*p*-aminophenol, aniline, and triaminopyridine are also excreted [9, 10]. Diminished renal function can extend this period as much as 7 hours [3].

Animal studies have shown that large doses of phenazopyridine can cause liver damage with central necrosis as well as toxic degeneration of renal collecting tubules [11]. In animal models, excessive doses of phenazopyridine often initially result in gastrointestinal distress accompanied by altered mental status followed by methemoglobinemia, jaundice, acute renal failure, and hemolytic anemia.

Methemoglobinemia is suspected to occur in phenazopyridine overdose due to either overproduction of oxidized hemoglobin or interruption of the reducing cascade [2, 6, 12]. This in turn oxidizes iron into its trivalent (ferric) form instead of its usual bivalent (ferrous) form. Thus, the affinity of hemoglobin to oxygen is increased, limiting oxygen delivery to tissues and causing hypoxia [13]. Methemoglobinemia does not appear in every case of phenazopyridine overdose; however, patients with G6PD deficiency are more vulnerable to it due to the loss of NADPH [6].

Jaundice can occur in phenazopyridine overdose either through hyperbilirubinemia from hemolytic anemia or independently via azo dye deposition in the skin. In the latter case, bilirubin, amylase, lipase, and transaminases remain within normal limits [3, 5]. It has also been theorized to occur secondary to acute hepatic injury in patients with elevated transaminases, although this mechanism has not been verified [14].

Acute renal failure is believed to occur via three possible mechanisms: direct phenazopyridine damage to the distal tubules, secondary to methemoglobinemia, or secondary to hemoglobinuria in hemolytic anemia. Direct toxic damage is most commonly observed on biopsies [2, 3]. Likewise, animal studies of triaminopyridine have shown vacuolization and necrosis of the distal tubules [15]. Certainly in this case direct toxicity appears to be the most likely cause of his renal failure, since neither hemolytic anemia nor methemoglobinemia were present. This acute tubular necrosis causes creatinine to peak between 3 and 10 days following ingestion, after hepatotoxicity, hemolysis, and methemoglobinemia have already manifested. Pigmented casts have been described in animal studies, in the urine of living patients, and in the biopsies of deceased patients [3, 11].

Hemolytic anemia in phenazopyridine overdose is one of the few described causes of anemia with bite cells and Heinz bodies [1, 5]. In fact, a case of phenazopyridine ingestion was one of the first in which bite cells were characterized and named [16]. The oxidant action of phenazopyridine overrides the HMP shunt, changing the oxidative state of hemoglobin and denaturing the globin chains. Subsequently, the chains precipitate with or without the heme groups to form Heinz bodies [17].

No standard treatment protocol has been established for the management of phenazopyridine overdose. Previous reports have described hydration, alkalinization of the urine, and diuresis if presenting before oliguria. Methylene blue is given for associated methemoglobinemia [2]. Later management has relied upon conservative and supportive measures with close observation in the hospital and a low threshold for dialysis for metabolic complications, such as in this case. Peritoneal dialysis has been tried as a possible therapy, although successful filtration of phenazopyridine could not be documented [18].

The significant limitation of this case report is the absence of a renal biopsy. However, phenazopyridine toxicity remains the most likely diagnosis. Onset of kidney injury, likely days before presentation, correlates with use of the medication. There were no other clear etiologies and no evidence of hemodynamic compromise in the days prior to presentation (although there was admittedly upon presentation).

In summary, this case demonstrates an unusual presentation possible with phenazopyridine overdose. Although the classic combination of acute renal failure, jaundice, hemolytic anemia, and methemoglobinemia is seen most frequently, individual components can occur without the others, regardless of the renal function of the patient. Why certain manifestations occur when others do not in any given patient has not been clearly established. However, the astute physician should be on the lookout for each component in any patient taking phenazopyridine.
